# Factors influencing physical activity participation among people living with or beyond cancer: a systematic scoping review

**DOI:** 10.1186/s12966-021-01116-9

**Published:** 2021-04-06

**Authors:** Sarah Elshahat, Charlene Treanor, Michael Donnelly

**Affiliations:** Centre for Public Health, Queen’s University Belfast, Institute of Clinical Sciences, Block B, Royal Victoria Hospital, Belfast, BT12 6BA UK

**Keywords:** Exercise, Physical activity, Cancer, Health belief model, Barriers, Facilitators

## Abstract

**Background:**

It has been posited that physical activity (PA) has the potential to improve health outcomes and the health-related quality of life of people living with or beyond cancer. Despite the well-documented health benefits of PA, there is a low level of PA among cancer patients. A systematic scoping review was conducted to investigate attitudes, perceptions, preferences and barriers vs. facilitators to cancer patients’ PA participation.

**Methods:**

A systematic search was performed across four automated databases (PubMed, Embase, PsycINFO and Medline) in keeping with the PRISMA guideline. All cancer types were included, and any age/gender groups were eligible. Both qualitative and quantitative studies were included. The Health Belief Model provided a conceptual framework for the conduct of the scoping review as well as guiding thinking to inform evidence-based interventions.

**Results:**

Ninety-eight articles were included in this review. Nearly half of the studies focused on mixed cancer sites; breast cancer was the most commonly examined cancer type (19%). Post-treatment was the most commonly investigated stage (33%), followed by studies of mixed stages of the cancer trajectory (27%), the acute treatment stage (23%) and pre-treatment stage (1%). Patient treatment stage was not reported in 16% of studies. Cancer patients reported positive attitudes to PA and recognized its benefits for health and wellbeing. Cancer-related side effects (e.g. fatigue) were a leading physiological barrier to PA participation, whereas effective symptom management techniques/tools acted as a powerful facilitator. Psychosocial barriers included low motivation and kinesiophobia, and perceived health benefits and social support/guidance by healthcare providers were significant facilitators. Inaccessible fitness facilities hindered cancer patients’ PA engagement though the availability of tailored amenities appeared to be a strong facilitator. PA preferences varied in terms of type, place, time, company and source of information and pointed to the need for individualized PA programs.

**Conclusions:**

There is a need for further research to identify barriers and facilitators to PA that are faced by patients with particular cancer types. Recommended PA promoting-strategies involve including exercise science professionals in healthcare teams and ensuring that fitness facilities are accessible.

**Supplementary Information:**

The online version contains supplementary material available at 10.1186/s12966-021-01116-9.

## Background

There is a growing body of evidence on the positive effects of physical activity (PA) engagement on cancer patients’ health outcomes, health-related quality of life (HRQoL) and survival rates [1, 2]. Cancer is the second major cause of death worldwide, which affected around 15 million of the global population in 2018 [3]. In Europe, cancer accounts for about 1.9 million annual deaths, 9% of which occurs in the UK [4, 5]. Furthermore, approximately 0.6 million Americans died of cancer in 2019 [6]. Sufficient PA engagement (i.e. weekly performance of ≥150 min moderate-intensity PA or ≥ 75 min vigorous-intensity PA) has been suggested to reduce cancer recurrence and improve HRQoL and survival rates among cancer patients [1]. A longitudinal study of 1432 breast cancer patients in the US showed that adequate PA participation was associated with reduced odds of cancer-specific mortality by 73% [7]. Similarly, a meta-analysis of six studies revealed that sufficient PA performance significantly reduced breast cancer-related deaths and disease recurrence among women with breast cancer [8]. Furthermore, a Canadian cohort study of 830 prostate cancer patients exhibited that participation in regular adequate post-diagnosis leisure PA significantly decreased cancer-specific and all-cause mortality [9]. Similar findings were also reported for other different cancer types [10–12].

Despite the well-documented benefits of PA for improving cancer patients’ health outcomes, adherence to the recommended PA guidelines among cancer patients appears to be poor [13]. For instance, two separate cross-sectional studies by Courneya et al. [14] and Speed-Andrews et al. [15] showed that about 70–80% of cancer survivors in Canada were physically inactive. Likewise, two American cross-sectional surveys of survivors of different cancers revealed a low self-reported adequate PA participation (around 30%) [16, 17]. A cross-sectional survey by Frikkel et al. [18] also revealed that only 22% of cancer patients in Germany were physically active. Only 15% of Australian cancer survivors engaged in sufficient PA compared to 45% of the general population [19].

This low PA level can in part be explained by potential barriers that minimize/hinder cancer patients’ PA engagement. For example, around 75% of participants in the cross-sectional studies by Fernandez et al. [20] and Romero et al. [21] reported that cancer therapy-related side effects acted as a barrier to PA participation. Furthermore, kinesiophobia was a common barrier to PA engagement among patients of different cancers [22, 23]. Lack of appropriate tailored facilities was also cited as a major barrier among cancer patients in different studies [24, 25].

The Health Belief Model (HBM) is a theoretical model that has widely been used in health promotion and disease prevention research to understand and predict individuals’ health behaviors, including PA and exercise [26–28]. The HBM proposes that individuals likely adopt a healthy behavior when they perceive their susceptibility to an illness/risk and its seriousness, and believe that the benefits to action outweigh the perceived barriers [29]. Cues to action (i.e. stimulus needed to prompt the adoption of health-related behaviors) and self-efficacy (i.e. confidence in one’s ability to adopt the health-related behavior) are two supplementary constructs later added to the HBM to enhance its efficacy [30]. Sheill et al. [31] employed the HBM to explore cancer patients’ views towards PA engagement. The study revealed that self-efficacy and perceived barriers, such as cancer-related side effects and inaccessible leisure facilities, were significant predictors of exercise behavior. Sheill et al. [31] concluded that more prompts are needed to increase cancer patients’ PA participation. These findings highlight the utility of the HBM in exercise-oncology research in providing a thorough understanding of the predictors of PA behaviors to inform the design of effective interventions.

Despite the importance of understanding the influences that affect cancer patients’ PA participation to effectively intervene, limited reviews were conducted in this area and those mainly focused on specific cancer types, particularly breast cancer [32, 33]. A comprehensive understanding of different factors that impact cancer patients’ PA engagement across various cancer types is crucial to direct future research and clinical practice. The HBM provided a conceptual framework for the conduct of this scoping review and the examination of the attitudes, preferences and influences that affect PA participation among patients of different cancer types in Western countries, and guiding thinking to inform effective, evidence-based interventions. Specific objectives of this review were to (1) explore cancer patients’ attitudes and perceived benefits and risks of PA participation, (2) investigate PA preferences among cancer patients, and (3) explore barriers and facilitators to cancer patients’ PA engagement.

## Methods

This study employed a scoping review methodology to examine the range and scope of the available literature on the investigated topic, producing a rigorous synthesis and disseminating the existing evidence to date. The five-stage scoping framework designed by Arksey and O’Malley [34] was employed alongside PRISMA guidelines for scoping reviews to maximize robustness [35].

### Research question formulation

A review question was articulated with a view to scoping broadly the relevant landscape of literature: *What are the perceptions, attitudes and factors influencing physical activity engagement among cancer patients?*

### Searching for relevant studies

A rigorous systematic search strategy was utilized to identify relevant records. Fifty-one search terms reflecting the review’s key concepts (cancer, physical activity and perceptions) were employed/combined through Boolean operators AND/OR to search four electronic databases (Embase, PubMed, PsycINFO and Medline) from inception to August 2020 (Additional file 1). The search was restricted to human studies and English language papers. A manual search of the Web, Google Scholar and relevant articles’ bibliographies was also implemented.

### Study selection

**Fig. 1:**
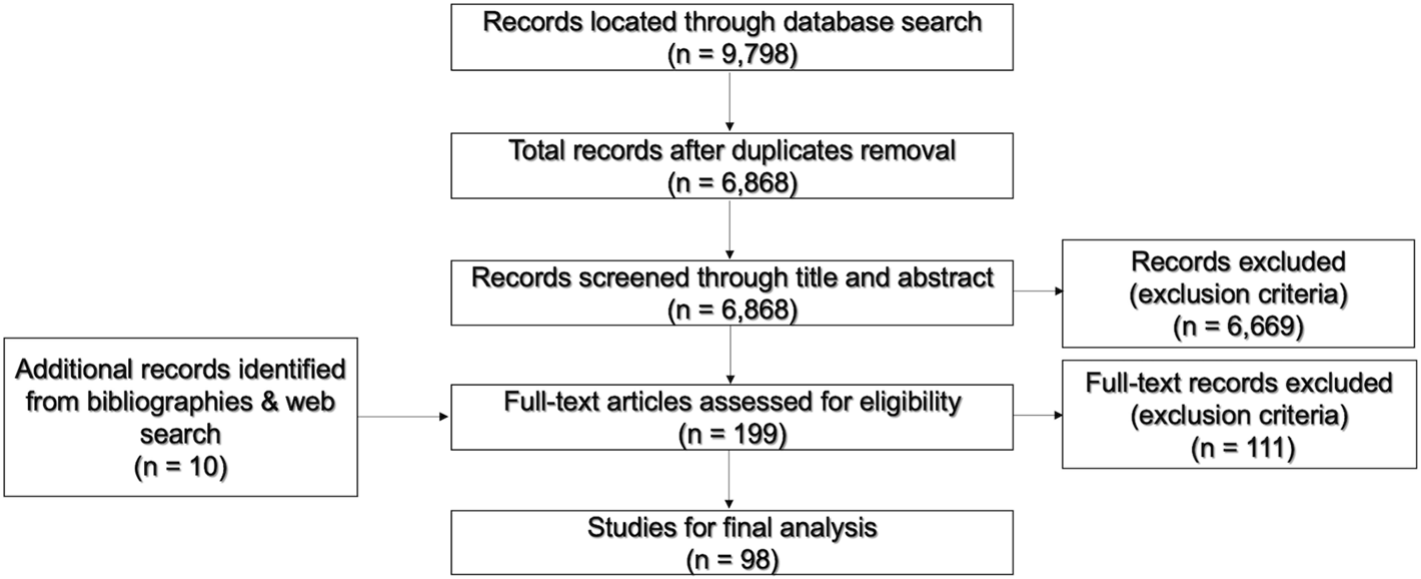
Flow diagram of the eligible studies

Studies were included if they examined attitudes, perceptions, PA preferences and/or barriers vs. facilitators to PA engagement among cancer patients. Only studies from the Western world (Europe, North America, Australia and New Zealand) were included in an effort to achieve a balance between reducing heterogeneity while being analytically thorough and pursuing the aims of the review. All cancer types were included, and any age/gender groups were eligible. Both qualitative and quantitative studies were included. Studies examining the impact of PA on cancer patients’ HRQoL, disease recurrence and survival rates were excluded. Papers assessing the cost-effectiveness of PA programs for cancer patients were not eligible. These inclusion/exclusion criteria were adopted to screen articles’ titles and abstracts as well as to entirely assess any potentially pertinent records (Fig. 1). Disagreements were addressed via frequent discussions between the authors.

### Data charting

An Excel-based data extraction tool was developed specifically for this review to facilitate the extraction of pertinent data from eligible records including authorship, country of study, research design, participants, cancer type and key findings.

### Collating, summarizing and reporting the results

**Fig. 2:**
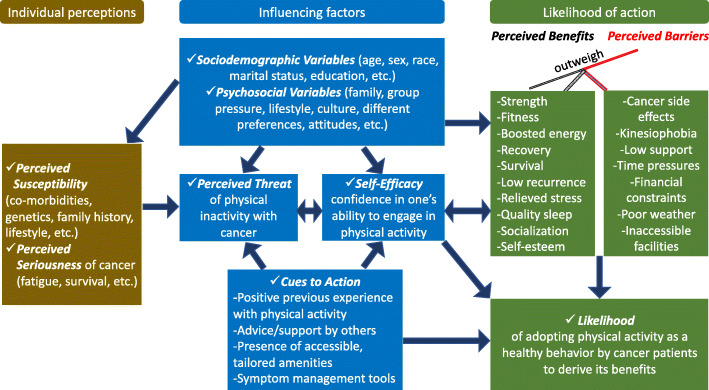
HBM-based conceptual model showing what predicts PA performance as a healthy behavior by cancer patients

A dual-stage analysis approach was adopted to synthetize the extracted evidence. First, data were subjected to a numerical synthesis to detect research gaps and enhance effective reporting. Second, three prime themes were generated to represent the extracted data in accordance with the review’s objectives. The HBM was used to develop a conceptual model that illustrated the main findings in terms of factors that influenced cancer patients’ performance and maintenance of PA as a healthy behavior (Fig. 2). The implications of the current review were also addressed in order to enhance the utility of the findings for future research, policy-making and clinical practice.

## Results

### Characteristics of the included studies

In total, 9798 articles were retrieved from the automated database search, in addition to 10 papers found through a manual search. Only 98 articles met eligibility criteria and were included for evidence synthesis (Fig. 1). Breast cancer was the most commonly investigated cancer site (19%), and 41% of studies examined mixed cancers (Additional file 2). One-third of studies focused on post-treatment survivors, and mixed treatment stages were examined in 27%. The US was the country where most studies were conducted (25%), followed by Canada (18%). Qualitative design was employed in 56% of the studies, and 38% used a cross-sectional survey design.

### Attitudes and perceived benefits and risks of PA

This theme involved 54 studies addressing cancer patients’ attitudes and perceived benefits and risks of PA participation. These were classified into two subthemes: 1) attitudes towards PA, and 2) perceived benefits and risks of PA.

#### Attitudes towards PA

Across cancer types, patients demonstrated positive attitudes to PA participation, expressing interest and willingness to engage in PA in order to derive its benefits [36–41]. For example, about half of participants in two mixed cancer cross-sectional studies by Quain et al. [42] and Blaney et al. [43] reported being interested in exercise opportunities and becoming physically active. Likewise, approximately 70–80% of mixed cancer participants in five separate quantitative and qualitative studies reported positive attitudes to PA, expressing a desire for PA program opportunities to increase their PA levels [44–48].

#### Perceived benefits and risks of PA

Cancer patients believed that PA was beneficial for their physical health and mental well-being (Table 1). Promoting health and recovery was the most commonly perceived physiological benefit reported by patients across cancer types and treatment stages in different qualitative studies. Cancer patients perceived that exercise had the potential to minimize cancer-related treatment side effects, particularly fatigue. This perceived health benefit was reported by around 90% of participants in three cross-sectional studies [43, 55, 71]. Enhancing cardiovascular fitness, boosting energy, improving muscle strength and managing body weight were also notable physiological benefits that were perceived by cancer patients at mixed treatment stages (Table 1). Approximately 90% of participants in cross-sectional studies by Mizrahi et al. [71] and Rogers et al. [55] believed that PA engagement built up their muscle strength. Across mixed cancer patient participants in qualitative studies, there was a common perception that exercise could prevent disease recurrence and improve their survival. Improved survival was reported as a perceived benefit by nearly 90% of participants in a mixed cancer cross-sectional study by Eng et al. [22].

**Table 1:** Perceived benefits and risks of PA (% patients citing the benefit/risk)

Cancer type	Country, no. of participants, treatment stage	Study design	Perceived benefits												Perceived risks			Reference
			Physiological benefits (A) Fitness, (B) Strength, (C) Promote health & recovery, (D) Boost energy, (E) Improve survival, (F) Prevent reoccurrence, & (G) Weight loss							Psychosocial benefits (H) Relieve stress, (I) Better state of mind, (J) Socialization, (K) Quality sleep, & (L) Self-esteem					(A) Fatigue, (B) Pain, & (C) Risk of injury			
			(A)	(B)	(C)	(D)	(E)	(F)	(G)	(H)	(I)	(J)	(K)	(L)	(A)	(B)	(C)	
Breast	Canada, 9, post-ttt	QI				+			+	+	+			+				[40]
	Canada, 8, during ttt	QI	+			+					+		+	+				[49]
	Canada, 12, mixed	FG	+		+ (25)	+ (8)	+ (17)			+	+ (17)			+	+	+ (17)	+	[50]
	Canada, 12, post-ttt	QI			+		+	+	+	+	+	+						[41]
	NZ, 20 women	FG	+									+						[51]
	Norway, 27 women, during ttt	FG	+		+			+		+	+			+				[52]
	Sweden, 16, on ttt	QI	+	+	+	+				+	+	+		+				[53]
	US, 60 women	QI	+ (28)			+ (15)				+ (5)	+ (18)							[54]
	US, 23 women, during ttt	CS		+ (91)	+ (91)	+ (78)			+ (91)	+ (87)	+ (83)	+ (52)		+ (78)		+ (30)	+ (4)	[55]
Colo-rectal	Australia,10, post-ttt	QI	+	+	+	+				+	+			+				[56]
	Canada, 600, post-ttt	CS	+ (70)		+ (66)	+ (61)	+ (58)	+ (41)	+ (52)	+ (54)	+ (44)							[57]
	Canada, 30, mixed	QI	+ (75)	+ (56)	+ (63)	+ (50)			+ (50)	+ (24)	+ (31)		+ (50)	+ (65)				[58]
	Sweden, 17 patients	QI			+													[59]
Endom-etrial	UK, 16 women, post-ttt	QI	+		+	+					+							[60]
Kidney	Canada, 482, post-ttt	CS	+ (26)	+ (25)	+ (22)	+ (23)			+ (49)				+ (3)	+ (24)				[61]
Leuke-mia	US, 6, post-ttt	QI			+	+				+		+	+	+				[62]
Lung	Australia, 7, post-ttt	FG	+	+	+					+	+							[63]
	Denmark, 19, post-ttt	QI	+	+	+	+					+			+				[64]
	US, 43, pre-ttt	CS	+ (13)	+ (67)	+ (18)	+ (13)									+ (48)	+ (29)	+(14)	[65]
Multiple myeloma	Australia, 24 patients, post-ttt	QI			+				+	+	+	+		+				[66]
Ovarian	Australia, 12, mixed	QI			+				+									[67]
Prostate	Australia, 18, post-ttt	QI			+	+			+	+	+	+		+				[68]
	Ireland, 20 men	QI	+		+	+			+		+							[31]
Various^a^	Australia, 9, on ttt	QI/FG		+	+						+			+				[69]
	Australia, 15, mixed	QI	+	+	+	+			+		+	+		+				[70]
	Australia, 102, post-ttt	CS		+ (88)	+ (96)	+ (44)		+ (63)		+ (68)		+ (61)						[71]
	Canada, 788 patients	CS				+ (76)	+ (90)											[22]
	Canada, 66, mixed	CS	+		+	+					+	+	+					[72]
	Canada, 13, post-ttt	QI		+	+	+		+		+	+	+		+				[73]
	England, 59, mixed	CS	+ (40)		+ (40)						+ (10)	+ (16)		+ (10)				[74, 75]
	Germany, 905 patients	CS	+ (58)		+ (68)									+ (61)				[76]
	Ireland, 41, mixed	FG	+		+	+		+		+	+	+	+	+				[77]
	Italy, 12 females, post-ttt	FG									+			+				[78]
	Netherlands, 13, post-ttt	QI	+	+		+				+	+	+		+	+			[79]
	Norway, 7, on ttt	QI		+	+				+	+	+							[80]
	NZ, 25, mixed stages	QI	+	+	+			+			+							[81]
	Sweden, 18, during ttt	FG	+	+	+	+			+		+	+		+	+	+		[24]
	UK, 6, post-ttt	QI	+	+	+	+			+	+	+	+		+				[82]
	UK, 19, post-ttt	QI			+			+	+		+							[37]
	UK, 16, mixed	QI		+	+	+								+				[83]
	UK, 26, mixed	FG			+				+	+	+			+	+	+	+	[84]
	UK, 456 patients	CS			+ (87)									+ (54)				[43]
	US, 20, during ttt	QI	+	+	+	+				+				+				[85]
	US, 25, post-ttt	FG			+			+										[86]

*Abbreviations*: *CS* cross-sectional, *FG* focus groups, *MM* mixed-method, *NZ* New Zealand, *PA* physical activity, *QI* qualitative interview, *ttt* treatment. ^a^ The study included various mixed cancer types

Improved state of mind was the most commonly reported perceived psychological benefit of PA participation among patients across cancer types and treatment stages (Table 1). Participants in qualitative studies by Hennessy et al. [82] and Husebø et al. [52] elaborated that PA enhanced their resilience and helped them focus on the positive aspects of life. Cancer patients also perceived that PA alleviated their stress and enhanced quality sleep. Around 85% of breast cancer patients in a cross-sectional study by Rogers et al. [55] believed that exercise reduced their psychological distress levels. Across cancer types, group exercising was perceived to reduce their isolation by providing socialization opportunities. Improved self-esteem and confidence in oneself were also among the perceived psychological benefits of PA.

Mixed cancer patients at different treatment stages perceived PA participation to be associated with increased risk of fatigue and exhaustion (Table 1). This perceived risk was reported by around 50% of lung cancer patient participants in a cross-sectional survey by Karvinen et al. [65]. Qualitative studies showed that pain and injury were also common perceived risks recognized by patients across cancer types. Risk of pain was reported by about 30% of lung and breast cancer patient participants in cross-sectional studies by Karvinen et al. [65] and Rogers et al. [55].

### PA preferences

Thirty-four studies explored the PA preferences of cancer patients and these were organized into three categories: 1) PA types and places for practice, 2) persons to provide information and time to start, and 3) company and time of the day.

#### PA types and places for practice

Walking was the most preferred type of PA cited by patients across cancer types during the treatment and post-treatment stages (Table 2). All breast cancer patient participants in a cross-sectional study by Rogers et al. [55] reported that walking was their favourite PA type. Similarly, around 75% of mixed cancer participants in cross-sectional studies by Blaney et al. [43] and Ross et al. [96] chose walking as their most preferred PA type. Qualitative studies revealed that swimming, cycling and yoga were also popular PA types among mixed cancer patients. Approximately, 60–70% of participants in cross-sectional studies of mixed cancer by Rogers et al. [55] and Ross et al. [96] identified swimming and cycling as their favourite PA types. Resistance weight and strength training were also commonly reported PA types. In their qualitative and quantitative studies, Owusu et al. [54] and Rogers et al. [55] found that half of breast cancer patient participants preferred resistance weight and strength training during the treatment and post-treatment stages. Other PA type preferences reported by patients across cancer types included gardening, jogging, ball sports, dancing, gymnastics and stretching.

**Table 2:** Preferences for PA types and places for practice (% patients reporting the preference)

Cancer type	Country, no. of participants, treatment stage	Study design	PA types												Places for PA practice				Reference
			(A) Walking, (B) Cycling, (C) Swimming, (D) Jogging, (E) Dancing, (F) Gardening, (G) Gymnastics, (H) Strength training, (I) Ball sports, (J) Yoga, (K) Resistance weight training, and (L) Stretching												(A) Home, (B) Outdoors/park, (C) Fitness center, and (D) Cancer center/hospital				
			(A)	(B)	(C)	(D)	(E)	(F)	(G)	(H)	(I)	(J)	(K)	(L)	(A)	(B)	(C)	(D)	
Brain	Canada, 31, on ttt	CS	+ (45)		+ (6)										+ (58)		+ (16)		[87]
	US, 106, mixed	CS	+ (51)										+ (44)		+ (26)		+ (9)	+ (6)	[88]
Breast	Canada, 524, post-ttt	CS	+ (51)							+ (27)		+ (36)		+ (36)	+ (32)		+ (51)	+ (5)	[89]
	Canada, 12, mixed	FG	+	+	+							+							[50]
	US, 60, post-ttt	QI	+ (52)	+ (37)			+ (23)			+ (80)			+ (52)						[54]
	US, 23, during ttt	CS	+(100)	+ (74)	+ (61)	+ (4)						+ (35)	+ (52)		+ (48)	+ (30)	+ (22)		[55]
	US, 160, during ttt	CS	+ (59)		+ (23)		+ (6)					+ (14)	+ (14)	+ (22)					[90]
Colo-rectal	Australia,10, post-ttt	QI	+		+				+	+	+		+						[56]
	Canada, 600, mixed	CS	+ (49)		+ (4)			+ (8)							+ (56)	+ (40)	+ (28)	+ (10)	[91]
	Netherlands, 15, mixed	QI	+	+			+	+							+				[92]
Endometrial	UK, 16, post-ttt	QI	+																[60]
Gynecologic	Canada, 239 patients	MM													+ (81)				[44]
Lung	Australia, 7, post-ttt	FG	+			+			+				+					+	[63]
	US, 43, pre-ttt	CS													+ (49)		+ (14)	+(23)	[65]
	US, 175, post-ttt	CS	+ (26)		+ (6)										+ (12)	+ (6)	+ (18)	+(10)	[38]
Multiple myeloma	Australia, 24, post-ttt	QI	+	+	+			+		+	+	+		+					[66]
Ovarian	Canada, 359, mixed	CS	+ (63)	+ (3)	+ (4)							+ (4)			+ (49)		+ (21)	+ (7)	[45]
Prostate	Australia, 18, post-ttt	QI	+			+		+	+		+	+							[68]
Testicular	Norway, 9, mixed	QI	+ (67)			+ (11)													[93]
Various^a^	Australia, 92, during ttt	CS	+ (68)	+ (4)	+ (6)							+ (5)	+ (9)		+ (53)		+ (15)		[94]
	Canada, 66, mixed	CS													+ (60)			+(32)	[72]
	England, 59, mixed	CS	+ (52)	+ (18)				+(73)				+ (18)	+ (17)						[74, 75]
	Germany, 155, mixed	CS	+ (45)	+ (45)	+ (45)				+ (13)		+ (20)	+ (13)							[95]
	Ireland, 41, mixed	FG			+ (15)					+ (66)		+ (3)			+ (68)				[77]
	Italy, 392, mixed	CS													+ (21)	+ (27)	+ (12)		[46]
	UK, 26, mixed stages	FG																+	[84]
	UK, 456 patients	CS	+ (73)		+ (31)					+ (34)		+ (26)		+ (31)	+ (14)		+ (23)	+(16)	[43]
	US, 20, during ttt	QI													+ (80)				[85]
	US, 162, mixed	CS	+ (73)	+ (64)	+ (58)	+ (36)	+ (35)				+ (31)				+ (91)	+ (58)	+ (64)		[96]

*Abbreviations*: *CS* cross-sectional, *FG* focus groups, *MM* mixed-method, *PA* physical activity, *QI* qualitative interview, *ttt* treatment. ^a^ The study included various mixed cancer types

Home and fitness centres were the most preferable places for PA practice among mixed cancer patients at different treatment stages (Table 2). About 80–90% of participants in three separate qualitative and quantitative studies reported that they would prefer performing PA at home [44, 85, 96]. Outdoor exercising was also a notable favourable option among patients of different cancer types and treatment stages. Finally, hospital setting was preferred by mixed cancer patients who recognized hospitals as the safest place for PA participation at different treatment stages (Table 2).

#### Persons to provide information and time to start

Studies showed that oncologists were the most preferable source of information among patients across cancer types, followed by physiotherapists and nurses (Table 3). Around 60–80% of mixed cancer patient participants in three different quantitative and qualitative studies preferred to receive PA information from oncologists [38, 46, 85]. Other preferable sources of PA information included family doctors and personal trainers.

**Table 3:** Preferences for PA information, timing and people to practice with (% patients reporting the preference)

Cancer type	Country, no. of participants, treatment stage	Study design	Persons to provide information					Time to start PA program				People to perform PA with			Time of the day			Reference
			(A) Oncologist, (B) Family doctor, (C) Nurse, (D) Physiotherapist, & (E) Personal trainer					(A) At diagnosis, (B) During ttt, (C) Immediately after ttt, & (D) 3–6 months after ttt				(A) Alone, (B) Family/friends, & (C) Other cancer patients			(A) Morning, (B) Afternoon, & (C) Evening			
			(A)	(B)	(C)	(D)	(E)	(A)	(B)	(C)	(D)	(A)	(B)	(C)	(A)	(B)	(C)	
Brain	Canada, 31, during ttt	CS										+ (52)	+ (26)	+ (7)	+ (16)	+ (29)	+ (7)	[87]
	US, 106, mixed	CS														+ (24)	+ (5)	[88]
Breast	Canada, 12, post-ttt	QI															+	[41]
	Canada, 524, post-ttt	CS	+ (14)	+ (17)	+ (14)	+ (35)		+ (21)	+ (14)	+ (37)	+ (18)	+ (50)	+ (21)	+ (14)	+ (21)	+ (17)	+ (13)	[89]
	NZ, 20 patients	FG															+	[51]
	US, 23, during ttt	CS						+ (39)	+ (13)	+ (30)	+ (13)	+ (35)	+ (26)	+(39)	+ (44)	+ (30)	+ (9)	[55]
Colo-rectal	Australia,10, post-ttt	QI							+	+	+						+	[56]
	Canada, 600, mixed	CS	+ (35)		+ (12)	+ (25)	+ (18)	+ (19)	+ (9)	+ (22)	+ (28)				+ (24)	+ (22)	+ (12)	[91]
	Netherlands, 15, mixed	QI	+	+											+ (53)			[92]
Endometrial	UK, 16 women, post-ttt	QI	+															[60]
Gynecologic	Canada, 239 women	MM								+ (63)		+ (79)						[44]
Lung	Australia, 7, post-ttt	FG							+	+							+	[63]
	US, 43, pre-ttt	CS	+ (23)	+ (19)	+ (2)	+ (12)	+ (7)	+ (16)	+ (47)	+ (21)	+ (7)				+ (23)	+ (40)	+ (7)	[65]
	US, 175, post-ttt	CS	+ (58)			+ (8)		+ (36)		+ (9)	+ (8)				+ (13)	+ (3)		[38]
Ovarian	Canada, 359, mixed	CS						+ (18)	+ (13)	+ (26)	+ (26)	+ (49)	+ (15)	+ (17)	+ (29)	+ (31)	+ (16)	[45]
Various^a^	Australia, 92, during ttt	CS	+ (18)		+ (17)	+ (9)		+ (41)	+ (21)	+ (35)								[94]
	Ireland, 41, mixed	FG	+	+	+												+	[77]
	Italy, 392, mixed	CS	+ (57)		+ (7)	+ (30)	+ (20)					+ (48)	+ (31)	+ (9)	+ (16)	+ (8)	+ (27)	[46]
	Norway, 7, during ttt	QI							+									[80]
	UK, 12, mixed stages	QI	+	+												+	+	[36]
	UK, 26, mixed stages	FG						+	+	+					+		+	[84]
	UK, 456, post-ttt	CS	+ (17)	+ (14)	+ (11)	+ (23)		+ (7)	+ (5)	+ (25)		+ (34)	+ (20)	+(15)	+ (15)	+ (19)	+ (38)	[43]
	US, 20, during ttt	QI	+ (80)															[85]

*Abbreviations*: *CS* cross-sectional, *FG* focus groups, *MM* mixed-method, *NZ* New Zealand, *PA* physical activity, *QI* qualitative interview. ttt: treatment. ^a^The study included various mixed cancer types

Overall, cancer patients preferred starting PA programs after finishing their cancer treatment (Table 3). For example, about half of participants with different cancers in four quantitative and qualitative studies reported “after treatment” as their most preferred time to start any PA programs [44, 45, 89, 91]. On the other hand, cross-sectional studies showed that starting PA programs immediately after cancer diagnosis and during treatment was preferred by about 20 and 10% of the patients, respectively [45, 89, 91] (Table 3).

#### Company and time of the day

Exercising alone was a commonly reported option by patients across cancer types and treatment stages (Table 3). Around half of mixed cancer participants in four cross-sectional studies admitted that they preferred exercising alone [45, 46, 87, 89]. On the other hand, about 20% of participants in a cross-sectional survey by Blaney et al. [43] chose to exercise either with family members or other cancer patients (Table 3).

Studies showed that morning was the favourite time of the day for mixed cancer patients to participate in PA, followed by the afternoon and the evening (Table 3). In their quantitative and qualitative studies, Rogers et al. [55] and Agasi-Idenburg et al. [92] noted that nearly half of participants preferred to engage in PA in the morning.

### Barriers and facilitators to PA participation

This theme comprised 82 studies addressing 13 barriers and 9 facilitators to PA engagement among cancer patients (Tables 4 and 5). Barriers and facilitators were organized into three main subthemes: 1) physiological factors, 2) psychosocial and cultural factors, and 3) economic and environmental influences.

**Table 4:** Barriers to PA participation among cancer patients (% patients reporting the barrier)

Cancer type	Country, no. of participants, treatment stage	Study design	Physiological		Psychosocial & cultural								Economic & environmental			Reference
			(A) Treatment side effects, & (B) Co-morbidities		(A) Low self-efficacy & motivation, (B) Low exercise discipline, (C) Kinesiophobia & (D) Not sporty, (E) Lack of social support, (F) Family responsibility, (G) Preference for other activities, & (H) Time pressures								(A) Financial issues, (B) Poor weather, & (C)Unavailable/ inaccessible facilities			
			(A)	(B)	(A)	(B)	(C)	(D)	(E)	(F)	(G)	(H)	(A)	(B)	(C)	
Breast	Canada, 9, post-ttt	QI	+	+	+			+	+	+	+	+	+	+	+	[40]
	Canada,160, during ttt	CS	+ (41)		+ (4)							+ (11)		+ (6)		[97]
	Canada, 8, during ttt	QI	+	+	+					+		+		+	+	[49]
	Canada, 12, mixed	QI	+		+				+	+	+	+			+	[77]
	Canada, 12, mixed	FG	+	+			+		+	+		+	+	+	+	[50]
	NZ, 20 women	FG			+			+	+					+	+	[51]
	Norway, 10, during ttt	QI	+		+											[98]
	Norway, 27, during ttt	FG	+						+	+		+				[52]
	Spain, 14, post-ttt	QI	+		+				+	+		+		+		[99]
	Sweden, 16, during ttt	QI	+		+					+		+			+	[53]
	UK, 83, during ttt	QI	+	+	+		+	+		+ (20)	+	+	+	+ (48)	+	[100]
	US, 23, during ttt	CS	+ (39)		+ (39)	+(52)	+	+	+ (26)	+ (22)	+ (26)	+ (39)	+ (22)	+	+ (22)	[55]
	US, 30 women, on ttt	QI	+						+		+					[101]
	US, 60 elderly, post-ttt	QI	+ (43)						+ (22)					+ (40)		[54]
Colo-rectal	Canada, 600, post-ttt	CS	+ (14)	+ (9)						+ (15)	+ (16)	+ (15)		+ (25)		[57]
	Canada, 69 survivors	RCT	+ (25)	+ (1)						+ (3)		+ (32)		+ (1)		[102]
	Netherland, 15, mixed	QI	+	+	+				+			+				[92]
	Spain, 30, during ttt	QI	+		+				+	+		+			+	[103]
	Sweden, 17 elderly	QI	+	+	+	+			+	+	+	+			+	[59]
	US, 30, mixed stages	QI	+	+	+										+	[104]
Endometrial	UK, 16, post-ttt	QI	+		+				+			+		+	+	[60]
Gynecologic	Canada, 239 patients	MM							+			+	+		+	[44]
Head/neck	England, 430, post-ttt	CS	+	+	+	+	+	+	+	+	+	+	+	+	+	[23]
Kidney	Canada, 482, post-ttt	CS	+ (20)	+ (24)	+ (14)					+ (19)		+ (22)		+ (15)	+ (8)	[61]
Lung	Australia, 7, post-ttt	FG	+	+	+	+	+	+	+			+	+	+	+	[63]
	Denmark, 19, post-ttt	QI	+	+												[64]
	France, 5, during ttt	QI	+	+	+		+		+					+		[105]
	UK, 28 patients	MM							+							[106]
	US, 43, pre-ttt	CS	+ (34)												+(16)	[65]
Lung & GI	US, 34 patients	QI	+	+	+		+	+		+	+	+		+	+	[107]
Lymphoma	US, post-ttt survivors	FG	+		+											[108]
Multiple myeloma	Australia, 24, post-ttt	QI	+		+		+									[66]
Ovarian	Australia, 95, mixed	CS	+ (38)		+ (26)	+ (36)	+ (12)	+(35)	+ (11)	+ (13)	+ (21)	+ (14)	+ (7)	+ (12)	+ (6)	[109]
	Australia, 95, mixed	CS	+ (38)	+	+	+ (33)		+(35)		+						[110]
	US, 10 women, post-ttt	QI	+	+						+		+		+	+	[111]
Prostate	Australia, 18, post-ttt	QI	+	+			+		+			+				[68]
	Australia, 14 men	QI	+	+	+	+			+	+		+	+			[112]
	England, 16 men, post-ttt	QI	+	+	+				+			+		+		[113]
	Ireland, 20 men	QI	+		+				+			+		+	+	[31]
Sarcoma	UK, 6, during ttt	QI	+		+				+							[114, 115]
Various^a^	Australia, 101, mixed	CS	+		+			+								[116]
	Australia, 20, on ttt	QI	+		+				+						+	[117]
	Australia, 10, post-ttt	CS	+ (52)		+ (14)		+ (9)		+ (5)		+ (39)	+ (34)		+ (15)	+ (13)	[71]
	Australia, 92, during ttt	CS	+ (51)		+											[94]
	Australia, 15, mixed	QI	+		+		+	+				+			+	[70]
	Australia, 9, during ttt	QI/FG	+		+				+						+	[69]
	Canada,66, mixed stages	CS	+				+				+	+			+	[72]
	Canada, 788 patients	CS	+ (41)	+ (4)	+ (30)		+ (13)		+ (14)	+ (25)	+ (19)	+ (30)		+ (26)	+ (10)	[22]
	Canada, 30, during ttt	CS	+ (74)		+ (82)		+ (26)		+ (60)							[20]
	Denmark, 33, during ttt	QI	+		+	+		+		+		+				[118]
	Denmark, 451, during ttt	CS	+ (74)						+ (14)			+ (14)	+ (13)		+ (15)	[48]
	England, 41, post-ttt	CS	+ (32)		+ (12)		+ (12)				+ (18)	+ (27)			+ (15)	[119]
	England, 59, mixed	CS	+ (48)	+ (22)	+ (18)	+ (18)	+ (18)	+(10)	+ (45)				+ (20)			[74, 75]
	Germany, 141 patients	CS	+ (77)	+ (85)	+ (50)		+ (27)	+(40)	+	+ (5)		+ (5)		+ (4)	+ (7)	[18]
	Ireland, 41, mixed	FG	+	+	+				+	+	+		+		+	[77]
	Italy, 12 females, post-ttt	FG	+				+		+			+		+	+	[78]
	NZ, 25, mixed stages	QI	+		+		+		+				+		+	[81]
	Norway, 7, during ttt	QI	+													[80]
	Sweden, 18, during ttt	FG	+	+	+				+			+			+	[24]
	UK, 19, post-ttt	QI	+	+					+			+		+		[37]
	UK, 456, post-ttt	CS	+ (36)	+ (37)	+ (27)		+ (20)							+ (26)	+ (26)	[43]
	UK, 6, post-ttt	QI	+		+		+			+	+	+		+		[82]
	UK, 12, mixed stages	QI	+	+					+							[36]
	UK, 1, mixed stages	QI	+				+	+							+	[83]
	UK, 26, mixed stages	FG	+		+		+		+			+	+		+	[84]
	US, 25, post-ttt	FG	+		+				+	+			+		+	[86]
	US, 162, mixed stages	CS	+ (34)		+ (36)	+ (24)			+ (35)			+ (24)				[96]
	US, 662, mixed stages	CS	+ (78)		+ (67)	+ (24)			+ (6)			+ (29)	+ (20)		+ (11)	[25]
	US, 20, post-ttt	QI	+	+	+				+			+	+		+	[39]
	US, 20, during ttt	QI	+		+											[85]
	US, 452 patients	CS	+		+		+		+	+	+	+		+	+	[120]
	US, 622 patients	CS	+ (78)		+ (68)	+ (65)										[21]
	US, 590 patients	CS	+	+							+ (8)	+ (21)				[42]
	US, 566 older adults	CS		+												[121]
	US, 640 patients	CS	+ (27)				+ (3)				+ (11)	+ (27)	+ (3)			[47]
	US, 13, mixed stages	QI	+		+		+		+						+	[122]

*Abbreviations*: *CS* cross-sectional, *FG* focus groups, *MM* mixed-method, *NZ* New Zealand, *PA* physical activity, *QI* qualitative interview, *ttt* treatment, *RCT* randomized controlled trial. ^a^ The study included various mixed cancer types

**Table 5:** Facilitators to PA participation among cancer patients (% patients reporting the facilitator)

Cancer type	Country, no. of participants, treatment stage	Study design	Physiological		Psychosocial & cultural					Economic & environmental		Reference
			(A) Feeling well, & (B) Symptom management strategies		(A) Positive previous experience, (B) Perceived benefits, (C) Exercise in routine (D) Social support and guidance, & (E)Companionship					(A) Affordable programs, & (B) Accessible/ tailored amenities		
			(A)	(B)	(A)	(B)	(C)	(D)	(E)	(A)	(B)	
Breast	Canada, 9 women, post-ttt	QI			+	+	+	+	+		+	[40]
	Canada, 8, during ttt	QI		+		+	+	+	+		+	[49]
	Canada, 12, mixed stages	QI				+			+			[77]
	New Zealand, 20 women	FG					+	+	+			[51]
	Norway, 27, during ttt	FG				+		+	+			[52]
	Spain, 14 women, post-ttt	QI				+		+	+		+	[99]
	Sweden, 16, during ttt	QI						+	+		+	[53]
	Sweden, 29, post-ttt	MM						+	+		+	[123]
	Sweden, 12, post-ttt	QI				+						[124]
	US, 15 women, post ttt	QI				+		+	+			[125]
	US, 60 women, post-ttt	QI						+ (50)	+ (97)		+ (20)	[54]
	US, 30 women, during ttt	QI						+			+	[101]
Colo-rectal	Canada, 600, post-ttt	CS	+ (10)						+ (13)		+ (12)	[57]
	Netherlands, 15, mixed	QI	+			+	+	+	+	+	+	[92]
	Sweden, 17 patients	QI	+	+		+		+			+	[59]
Endometrial	UK, 16 women, post-ttt	QI				+		+	+			[60]
Gynecologic	Canada, 239 survivors	MM				+ (50)		+ (50)	+	+	+	[44]
Kidney	Canada, 482, post-ttt	CS							+ (47)		+ (7),	[61]
Lung	Australia, 7, post-ttt	FG			+			+	+		+	[63]
	Denmark, 19, post-ttt	QI				+	+	+			+	[64]
	France, 5 patients	QI				+		+	+			[105]
	US, 43, during ttt	CS	+ (15)				+ (11)	+ (15)			+ (37)	[65]
Lung & GI	US, 34 patients	QI			+	+	+	+	+			[107]
Lymphoma	US, N/A, post-ttt	FG						+			+	[108]
Prostate	Australia, 18 men, post-ttt	QI						+	+			[68]
	Australia, 14 men	QI		+				+	+			[112]
	England, 16 men, post-ttt	QI		+		+		+	+		+	[113]
	Ireland, 20 men	QI						+				[31]
Various^a^	Australia, 15, mixed stages	QI				+		+	+			[70]
	Australia, 9, during ttt	QI/FG					+	+		+	+	[69]
	Australia, 102, post-ttt	CS			+	+ (99)		+ (97)	+ (81)		+	[71]
	Australia, 20, during ttt	QI						+			+	[117]
	Canada, 30, during ttt	CS			+ (63)	+ (37)	+	+ (32)	+		+ (42)	[20]
	Denmark, 33, during ttt	QI		+		+	+	+	+			[118]
	Germany, 905 patients	CS				+	+		+			[76]
	Ireland, 41, mixed stages	FG						+	+		+	[77]
	Italy, 12, post-ttt	FG			+	+	+	+	+		+	[78]
	New Zealand, 25, mixed	QI						+	+		+	[81]
	Sweden, 18, during ttt	FG		+	+			+	+		+	[24]
	UK, 26, mixed stages	FG				+	+	+	+		+	[84]
	UK, 456, post-ttt	CS		+				+	+		+	[43]
	UK, 19 patients, post-ttt	QI						+	+		+	[37]
	UK, 12, mixed stages	QI		+				+	+			[36]
	UK, 16, mixed stages	QI						+			+	[83]
	US, 25, post-ttt	FG				+	+	+	+		+	[86]
	US, 20, post-ttt	QI						+	+	+	+	[39]
	US, 13, mixed stages	QI				+		+			+	[122]

*Abbreviations*: *CS* cross-sectional, *FG* focus groups, *MM* mixed-method, *N/A* non-available, *PA* physical activity, *QI* qualitative interview, *ttt* treatment. ^a^ The study included various mixed cancer types

#### Physiological factors

Studies revealed that cancer and its related treatment’s side effects acted as a significant physiological barrier to PA participation among patients across cancer types and treatment stages (Table 4). In five cross-sectional studies, about 70–80% of mixed cancer patient participants at different treatment stages reported that cancer therapy-related adverse effects hindered their PA engagement [18, 20, 21, 25, 48]. The most common and significant side effects reported by mixed cancer patients were fatigue, gastrointestinal issues and joint pain. Other additional adverse effects were site-specific, such as urinary incontinence (prostate cancer), upper-limb movement issues (breast cancer) and feeding tube limitations (head/neck cancer). Qualitative studies showed that the presence of co-morbidities was a major barrier to PA among mixed cancer patients at different treatment stages (Table 4). The most prevalent co-morbidities reported by cancer patients included arthritis, diabetes and heart disease. Cross-sectional studies by Bluethmann et al. [121] and Frikkel et al. [18] showed that comorbidity was a significant negative predictor of PA levels among mixed cancer patients.

On the other hand, cancer patients in different quantitative and qualitative studies reported that feeling well (i.e. having no physical symptoms and pain) facilitated their PA participation (Table 5). The presence of effective cancer symptom management strategies was a significant facilitator to PA participation among patients across cancer types and treatment stages. For example, mixed cancer patients in qualitative studies by Karlsson et al. [59] and Swan et al. [36] explained that the presence of tools/products that help minimize pain during exercising (e.g. TheraBand) would help enhance their PA participation. Furthermore, prostate cancer patients in a qualitative study by Hackshaw-McGeagh et al. [113] narrated that the availability of well-fitting, comfortable pads would enable them to engage in PA by managing urinary incontinence issues.

#### Psychosocial and cultural factors

Quantitative and qualitative studies showed that low self-efficacy and motivation and limited exercise discipline were common barriers to PA engagement among cancer patients across treatment stages (Table 4). About 70–80% of mixed cancer participants in cross-sectional studies by Fernandez et al. [20], Romero et al. [21, 25] and Frikkel et al. [18] reported that lack of motivation limited their PA participation. Cancer patients linked their low motivation to feelings of embarrassment and concerns about appearance when exercising in public. Kinesiophobia was a major barrier to PA engagement among cancer patients who were concerned about fall and injury. Cancer patients, moreover, cited “never been active” and “being not sporty” as common barriers to PA participation. In their cross-sectional study, Frikkel et al. [18] noted that not being active prior to diagnosis was a significant positive predictor of physical inactivity among cancer patients. Lack of social support was a key barrier to PA participation among patients across cancer types in separate quantitative and qualitative studies (Table 4). Cancer patients delineated that discouragement by family members and limited support/guidance by clinicians impeded their PA engagement [69, 103]. Family responsibility was a significant barrier to PA participation among cancer patients, particularly women who prioritized their family/children over self. Cancer patients preferred spending their free time with families, co-engaging in social activities rather than exercising [101]. Qualitative and quantitative studies revealed that time pressure was a common barrier to PA engagement. Mixed cancer patients explained that work commitments and cancer-related medical appointments minimized time available for exercising [52, 63, 100].

Qualitative and quantitative studies exhibited that perceived health benefits and positive previous experiences with exercise (exercise-related improvement in cancer symptoms) were strong facilitators to PA participation (Table 5). A cross-sectional study by Mizrahi et al. [71] revealed that 99% of the cancer patient participants found perceived health benefits to be useful for enhancing their PA participation. Furthermore, having exercise in one’s routine facilitated PA engagement among cancer patients. Mixed cancer qualitative and quantitative studies showed that social support was a powerful facilitator to PA participation during and post-treatment stages (Table 5). This included having supportive family and friends and helpful/encouraging healthcare professionals that provide sincere guidance on exercise performance. Companionship was a common facilitator reported by patients across cancer types. In their qualitative and quantitative studies, Owusu et al. [54] and Mizrahi et al. [71] found that group exercising with significant others and/or other cancer patients who face similar challenges was a major facilitator among 80–95% of the participants.

#### Economic and environmental factors

Financial issues represented a major economic barrier to PA participation among patients across cancer types (Table 4). Cancer patients in qualitative and quantitative studies by Hefferon et al. [100] and Catt et al. [74] explained that not affording gym memberships hindered their PA participation. Poor weather was a common barrier to PA engagement among cancer patients. Qualitative and quantitative exercise-oncology research showed that inaccessible facilities represented a significant barrier to PA participation among cancer patients across cancer types and treatment stages. Cancer patients detailed that lack of disabled-friendly spaces (e.g. privacy in changing rooms), limited availability of cancer-specific exercise services and inaccessible parking areas hindered their PA engagement [24, 51, 72, 77, 86, 100].

Qualitative studies showed that availability of affordable PA programs was a common facilitator to PA participation among cancer patients (Table 5). The presence of accessible, tailored amenities was cited as a significant facilitator to PA participation in different quantitative and qualitative studies. Mixed cancer patients reported that the availability of facilities with tailored and individualized PA programs would enable them to be physically active [83, 84, 122].

## Discussion

This scoping review examined attitudes, perceptions, preferences and barriers vs. facilitators to PA participation among cancer patients to direct future research and inform the development of tailored PA programs. Nearly half of studies investigated mixed cancers, and breast cancer was the most commonly examined cancer type (19%), highlighting a research gap in other cancer sites particularly cancers with high incidence and prevalence. For example, in 2018, globally, lung cancer was the most prevalent cancer type (12.3%) and the most common cause of cancer death (1.8 million deaths) [3]. This review showed that the US is the lead country in exercise-oncology research. Most studies (96%) employed either qualitative or cross-sectional quantitative research designs which are appropriate for capturing the views of patients.

The HBM aided the analysis of the results of our review and helped to conceptualize and illustrate predictor variables and influencers of PA participation among cancer patients (Fig. 2). Perhaps unsurprisingly, most if not all patients perceived the seriousness of the disease and felt susceptible and vulnerable though these perceptions tended to be tempered by cancer patients. Patients appeared likely to adopt PA as a healthy behavior when they believed that the benefits associated with PA outweighed any perceived barriers, particularly when a patient felt self-efficacious and there were positive cues to PA engagement and even more so when physical inactivity was a perceived threat to recovery. According to our model-guided analysis, the likelihood of cancer patients engaging in physical activity is not a simple linear or sequential process - a set of reciprocal relationships appear to exist between self-efficacy, perceived threat, perceived barriers and benefits; and sociodemographic and psychosocial variables individually and collectively exert influences on this set of relationships and on the likelihood of PA engagement by cancer patients.

Our study demonstrated that most cancer patients showed positive attitudes to PA and were motivated to enhance their PA levels, however, they faced numerous barriers that hindered their PA participation. Providing effective cues to action (e.g. inspiring stories of patients who adopted PA behaviors) may help address any negative or neutral attitudes towards PA among cancer patients [31]. Cancer and its related treatment’s side effects acted as a physiological barrier to PA among patients across cancer types. Fatigue is a common symptom among cancer patients described as a feeling of exhaustion that often limits cancer patients’ ability to participate in PA [126]. Cancer-related fatigue can be attributed to different factors, including fatigue-triggering cancer-related cytokines and the destruction of healthy cells by cancer treatment. PA has been suggested to help combat fatigue among cancer patients [69]. A Cochrane review of exercise interventions for managing cancer-related fatigue found evidence that exercise/PA was effective during and after cancer treatment [127]. Our review showed that perceived health benefits of PA for fatigue management encouraged cancer patients to participate in PA.

Low motivation owing to self-consciousness about appearance limited cancer patients’ PA participation. Cancer patients may have concerns about being judged or getting their appearance criticized by others when exercising [86]. The availability of group exercise opportunities with other cancer patients appeared to help address the low motivation barrier, fostering cancer patients’ PA participation. Exercising with other cancer patients can help elicit a sense of comfort/belonging, allowing for peer support and enhancing cancer patients’ motivation to participate in PA [92]. One significant psychosocial barrier revealed in this review was low social support and encouragement by significant others and healthcare professionals who provided limited PA advice and guidance. The main focus on therapeutic treatment and discharge planning in cancer settings with little attention given to PA education and exercise-based rehabilitation is a common issue in healthcare services in Western countries [103]. Healthcare providers in Europe and the US reported that time pressure in clinics limited their ability to support and guide cancer patients on PA engagement for improving their health outcomes [85, 128]. Given the suggested health benefits of PA for improving cancer patients’ health outcomes and survival, exercise education and rehabilitation should be an integral part of cancer services.

Inaccessibility of exercise facilities was a significant environmental barrier to cancer patients’ PA participation. Indeed, inaccessible fitness facilities is a major issue that hinders PA engagement among people with disabilities [129]. A study by Rimmer et al. [130] utilized the Accessibility Instrument Measuring Fitness and Recreation Environments tool to assess the accessibility of 227 fitness facilities in 10 American states based on 15 varied criteria, including access routes, parking, professional support and policy. Rimmer et al. [130] noted that most fitness facilities exhibited low accessibility scores across the 15 investigated criteria, revealing a pressing need for improving fitness facilities’ accessibility to enhance PA participation among people with disabilities. Similar findings were reported in a systematic review of 14 studies by Cadler et al. [131]. The availability of accessible/tailored amenities appeared to be a significant facilitator to cancer patients’ PA participation. Our review showed varied PA preferences among cancer patients across the type, place, time, source of information and company domains. For example, while some patients considered home or fitness centers as the most favourable place to exercise, others preferred exercising in hospitals. The preference for exercising outside healthcare settings may in part be explained by a desire to restore a sense of normality, whereas the choice of hospitals as a place to exercise can be attributed to the need for feelings of health safety [89]. These findings highlight the need for individualized PA programs that are designed to best serve each patient’s needs.

## Strengths and limitations

This scoping review investigated perceptions, preferences and factors influencing PA participation among patients diagnosed with any cancer types to identify gaps in research. Our review produced a HBM-guided conceptual model that represents the set and pattern of factors that influence cancer patients’ uptake of PA behavior. A strength of our review was that it adopted a rigorous systematic search strategy to address its objectives. The results of the qualitative and quantitative study designs ‘triangulated’ or concurred, overall, thereby adding to the synthesis of the available evidence and adding to the confidence about the believability of the review findings. It is important to note that this review included only English language papers and solely studies from Europe, North America, Australia, and New Zealand which may limit the review’s findings in terms of the extent to which they might be transferable to low- and middle-income countries, where resources are constrained and the context, culture, organisation, management and delivery of healthcare are different. This review did not involve a quality appraisal of the included studies, in keeping with scoping review methodology guidelines by Arksey and O’Malley [34], and in order to capture a wide range of types of evidence and study designs and identify gaps in research on the study topic.

## Conclusions and recommendations

Most exercise-oncology research focused on mixed cancer patients, and breast cancer was the most commonly investigated cancer type. Cancer patients exhibited positive attitudes towards PA participation and perceived PA to be beneficial for health and wellbeing. Key barriers to PA engagement among cancer patients included treatment-related side effects, low motivation, kinesiophobia, low social support, time pressures and inaccessible fitness facilities, whereas effective symptom management strategies, perceived health benefits, social support and guidance, and availability of tailored amenities were powerful facilitators. PA preferences among cancer patients varied in terms of type, place, time, company and source of information, underscoring the need for personalized PA programs that are developed to best meet patients’ needs.

Based on this review’s findings, we recommend the implementation of mixed-methods research to provide a robust and comprehensive understanding of perceptions, preferences and factors that influence PA participation among cancer patients. Future mixed cancer research should consider analysis by cancer site, and more cancer type-specific studies should be carried out to identify barriers and facilitators to PA that may be pertinent to particular cancer types. There is a need, too, for RCT designs that will determine cancer-specific side effects and inform effective symptom management strategies and appropriate, individualized PA prescriptions and programs. We recommend employing conceptual models such as the HBM framework in future exercise-oncology research to gain as comprehensive as possible understanding about the complex pattern of relationships between variables that predict or influence the adoption of PA as a healthy behavior by cancer patients and to inform the design of necessarily tailored interventions.

Regarding policy and practice, we recommend some actions for consideration by policymakers and commissioners to enable cancer patients to increase their PA participation. These involve the inclusion of exercise science professionals in healthcare professional teams to help develop tailored PA services in canter settings. Furthermore, healthcare providers should be encouraged to provide effective exercise education, including support and guidance on PA engagement, to cancer patients. We also propose the implementation of exercise-based rehabilitation as an integral part of cancer treatment settings. Innovative medical healthcare products and technologies that are mainly designed to help manage cancer-related side effects and enhance cancer patients’ PA participation are recommended. Finally, community-based fitness facilities are encouraged to enhance their accessibility to serve as a potential health promotion place for people with disabilities, including cancer [131].

## Supplementary Information

**Additional file 1.** The concepts and search terms run in the automated databases.

**Additional file 2.** Characteristics of the 98 included studies.

## Data Availability

All data analyzed in this review are presented in the published article and its additional files 1 and 2.

## Abbreviations

HBM: Health belief model
HRQoL: Health-related quality of life
PA: Physical activity
