# Supplementary material for: Factors influencing physical activity participation among people living with or beyond cancer: a systematic scoping review
Source: Int J Behav Nutr Phys Act. 2021 Apr 6;18:50. doi: 10.1186/s12966-021-01116-9 (PMC8025326; doi:10.1186/s12966-021-01116-9)
Supplement: Supplementary file 1 — Additional file 1. The concepts and search terms run in the automated databases. [file 12966_2021_1116_MOESM1_ESM.docx]

**Additional file 1. The search terms run in the automated databases**

| **Concept 1** | **Concept 2** | **Concept 3** |
| --- | --- | --- |
| cancer* OR “cancer therap*” OR “cancer treatment*” OR  “chemotherap*” OR “cancer surger*” OR neoplasm* OR malignan* OR carcinoma* OR tumo#r* | “physic* activ*” OR PA OR exercis* OR walk* OR “active liv*” OR bicycl* OR cycl* OR active lifestyle*” OR exert* OR “physic* ﬁt*” OR sport* OR jog* OR swim* OR “weight lift*” OR “strength train*” OR “resistance train*” OR “circuit weight train*” OR “aerob* train*” OR “physical education” OR “physical-ﬁtness” OR “training” | percept* OR attitude* OR perspective* OR belief* OR believ* OR perceiv* OR prefer* OR view* OR knowledg* OR Behavi* OR experience* OR accepta* OR barrier* OR facilitator* OR motivator* OR motiv* OR enabler* OR “qualitative stud*” OR “qualitative research*” OR interview* OR “focus group*” |
