# Supplementary material for: Factors influencing physical activity participation among people living with or beyond cancer: a systematic scoping review
Source: Int J Behav Nutr Phys Act. 2021 Apr 6;18:50. doi: 10.1186/s12966-021-01116-9 (PMC8025326; doi:10.1186/s12966-021-01116-9)
Supplement: Supplementary file 2 — Additional file 2. Characteristics of the 98 included studies. [file 12966_2021_1116_MOESM2_ESM.docx]

**Additional file 2. Characteristics of the included studies**

| **Study category** | **Number of studies n (%)** |
| --- | --- |
| **Country of authorship** | |
| US | 24 (25%) |
| Canada | 18 (18%) |
| Australia | 14 (14%) |
| New Zealand | 2 (2%) |
| UK | 16 (16%) |
| Europe | 24 (25%) |
| **Study design** | |
| Cross-sectional | 38 (38%) |
| Qualitative interviews | 41 (41%) |
| Focus groups | 10 (10%) |
| Mixed qualitive interview and focus groups | 5 (5%) |
| Mixed-method | 3 (3%) |
| RCT | 1 (1%) |
| **Cancer site** | |
| Mixed | 41 (41%) |
| Breast | 19 (19%) |
| Colo-rectal | 9 (9%) |
| Lung | 7 (7%) |
| Ovarian | 5 (5%) |
| Prostate | 4 (4%) |
| Gynecologic | 2 (2%) |
| Brain | 2 (2%) |
| Sarcoma | 2 (2%) |
| Kidney | 1 (1%) |
| Head/neck | 1 (1%) |
| Multiple myeloma | 1 (1%) |
| Endometrial | 1 (1%) |
| Testicular | 1 (1%) |
| Leukemia | 1 (1%) |
| Lymphoma | 1 (1%) |
| **Stage of cancer treatment** | |
| Pre-treatment | 1 (1%) |
| During treatment | 23 (23%) |
| Post-treatment | 32 (32%) |
| Mixed stages | 26 (26%) |
| Not clearly defined | 16 (16%) |
| **Record type** | |
| Journal article | 91 (93%) |
| Conference proceeding | 7 (7%) |
